# Clinical Efficacy of Adjuvant Treatment of Primary Nephrotic Syndrome in Pediatric Patients with Chinese Medicine

**DOI:** 10.1155/2022/1516633

**Published:** 2022-01-25

**Authors:** Hongjie Wu, Lin Zhang, Qing Liu, Baofeng Ren, Jun Li

**Affiliations:** ^1^Department of Pharmacy, Yantaishan Hospital, Yantai 26400, China; ^2^Department of Health Materials Management, Dongying People's Hospital, Dongying Hospital Affiliated to Shandong Provincial Hospital Group, Dongying 257091, China; ^3^Department of Traditional Chinese Medicine, Zhangqiu District People's Hospital, Jinan 250200, China; ^4^Medical Insurance Department, Zhangqiu District People's Hospital, Jinan 250200, China; ^5^Department of Pharmacy, The Affiliated Hospital of Shandong University of TCM, Jinan 250011, China

## Abstract

**Objective:**

Current study aimed to investigate the benefits of adjuvant therapy with traditional Chinese medicine on the pediatric primary nephrotic syndrome.

**Methods:**

A total of 455 patients with PNS admitted to our hospital from January 2010 to January 2019 were divided into the traditional Chinese medicine group (*n* = 217) and the control group (*n* = 238). The control group received conventional Western medical treatment, and the traditional Chinese medicine group was treated with traditional Chinese medicine supplemented with Western medical treatment. The differences in remission rate, recurrence rate, and recurrence-free survival between the two groups were evaluated.

**Results:**

The differences in clinical parameters between the two groups were not statistically significant. Compared with the control group, adjuvant treatment with traditional Chinese medicine increased the clinical remission rate (*p*=0.037), decreased the relapse rate (*p*=0.015), prolonged relapse-free survival (*p* ≤ 0.01), and was an independent protective factor for relapse-free survival in children with PNS (HR = 0.55, 95% CI 0.49–0.63, *p* ≤ 0.01). In a subgroup analysis of the traditional Chinese medicine formulations, Yuebi Jiazhu Tang, Ganlu Xiaodu Dan, and Yupingfeng granules significantly reduced the risk of recurrence in children (*p* ≤ 0.01, *p* ≤ 0.01, *p*=0.003).

**Conclusion:**

Adjuvant treatment of pediatric primary nephrotic syndrome with traditional Chinese medicine could benefit the children.

## 1. Introduction

Pediatric nephrotic syndrome can be divided into primary nephrotic syndrome and secondary nephrotic syndrome according to the cause, among which primary nephrotic syndrome (PNS) accounts for more than 90% of nephrotic syndromes [1]. PNS is caused by a variety of causes of elevated glomerular basement membrane permeability, and a large number of plasma proteins pass through and are excreted in the urine. Clinical manifestations are mainly characterized by massive proteinuria, hypoproteinemia, and hyperlipidemia [2]. Currently, glucocorticoids are the drugs of choice for the treatment of primary nephrotic syndrome in pediatric patients, and they are administered for a longer period time to correct the inflammatory and immune response of the body and affect the permeability of the glomerular basement membrane, thus exerting a diuretic effect and eliminating proteinuria. Clinically, children can be classified into hormone-sensitive and hormone-resistant nephrotic syndrome according to their response to hormone therapy [3]. In China, even if children are sensitive to initial hormone therapy, 80–90% of them still relapse, of which 25–43% are frequent relapses or hormone-dependent [4]. Hormone-resistant, hormone-dependent, and frequently relapsing nephropathies require treatment with hormones in combination with immunosuppressive drugs (e.g., cyclosporine A, tacrolimus, cyclophosphamide, tacrolimus, and rituximab) [5–7]. Long-term use of glucocorticoids also leads to an increased risk of adverse effects (e.g., osteoporosis, growth disorders, infections, and obesity) [8], and side effects such as nephrotoxicity, hypertension, and diabetes may occur with calcineurin inhibitors [9].

Therefore, the combined treatment of nephrotic syndrome with Chinese and Western medicine has become a hot topic in the field of pediatric nephrotic syndrome research to achieve the best outcome for children with nephrotic syndrome, effectively control proteinuria, reduce recurrence, smoothly withdraw immunosuppressive drugs, and promote remission. There is increasing evidence that plants used in traditional Chinese medicine (TCM) and compounds isolated from medicinal plants have beneficial effects and that TCMs can improve clinical remission rates and reduce the frequency of relapses and infections in children with PNS as an adjunct to Western medical treatment [10]. Therefore, this retrospective study aimed to investigate the efficacy of adjuvant therapy with traditional Chinese medicine in children with primary nephrotic syndrome.

## 2. Clinical Data and Methods Clinical Data

### 2.1. Clinical Data

This study was a retrospective study of children hospitalized in the Affiliated Hospital of Shandong University of TCM with newly diagnosed primary nephrotic syndrome from January 2010 to January 2019. The study was approved by the Ethics Committee of the Affiliated Hospital of the Shandong University of TCM. Inclusion criteria were as follows: meeting the diagnostic criteria for pediatric primary nephrotic syndrome, age 1–14 years, and follow-up period >1 year. Exclusion criteria were as follows: secondary nephrotic syndrome, such as hepatitis B virus-associated nephritis, lupus nephritis, and congenital and hereditary nephrotic syndrome; other renal systemic diseases causing edema or proteinuria; not receiving conventional Western medical treatment; and incomplete clinical data and follow-up data. A total of 455 children were finally eligible for inclusion and were divided into the group receiving conventional Western medical treatment (hereafter referred to as the control group) and the group receiving combined Western + Chinese medicine treatment (hereafter referred to as the TCM group).

### 2.2. Methods

Interventions: children in the control group received conventional Western medical treatment. In addition to receiving conventional treatment of Western medicine, the children in the TCM group also had to have medical records of using TCM (the course of treatment was at least 1 month). The common prescriptions used in the treatment of pediatric PNS according to the TCM evidence are Yuebi Jiazhu Tang (ephedra 12 g, gypsum 25 g, fresh ginger 9 g, licorice 6 g, largehead atractylodes rhizome 12 g, and Chinese date 15, decocted by the TCM pharmacy of our hospital), Ganlu Xiaodu Dan (SFDA approval number Z33020075; talc, baical skullcap root, virgate wormwood herb, grass leaf sweet flag rhizome, akebia stem, blackberry lily rhizome, cardamon fruit, weeping forsythia capsule, tendril leaf, cablin patchouli herb, and peppermint), and Yupingfeng granules (SFDA approval number Z10930036; milkvetch root, largehead atractylodes rhizome, and divaricate Saposhnikovia root).

### 2.3. Observation Index

Remission: urine protein creatinine ratio (uPCR) < 0.2 mg/mg (<20 mg/mmol) or morning urine protein <+ for 3 d.

Relapse: uPCR ≥2.0 mg/mg (200 mg/mmol) or morning urine protein ≥+++ for 3 d [11].

Relapse-free survival: the last follow-up period shall prevail for children without recurrence.

### 2.4. Statistical Analysis

SPSS 25.0 software was used for statistical analysis. $\bar{x}$ ± *s* was used for the statistical description of measurement data, the unpaired *t*-test was used for comparison between groups, and the rank-sum test was used for data that did not conform to normal distribution. The statistical description of the usage rate of the count data was performed, and the *χ*^2^ test was used for comparison between groups. Kaplan–Meier survival curves and log-rank tests were used to compare the differences in relapse-free survival between children in the TCM and control groups. Univariate and multifactorial Cox analyses were used to elucidate the risk factors affecting recurrence in children with PNS. *P* < 0.05 was considered a statistically significant difference.

## 3. Results

### 3.1. Baseline Characteristics

A total of 455 children were included in the study, including 238 in the control group, 183 males and 55 females, with a mean age of (4.93 ± 2.25) years, and 217 in the TCM group, 164 males and 53 females, with a mean age of (5.05 ± 2.34) years. Baseline characteristics were compared between the two groups, including gender, age, clinical classification, 24 h urine protein quantification, serum albumin level, blood creatinine, blood urea nitrogen, serum cholesterol, IgG, IgM, IgA, and ESR. There were no statistically significant differences in these parameters between the two groups (*p* > 0.05, Table 1).

### 3.2. Remission Rate and Recurrence Rate

In the TCM group, 198 children were in clinical remission after treatment and 19 children were not in remission; in the control group, 202 children were in clinical remission after treatment and 36 children were not in remission, and the remission rate in the TCM group was significantly higher than that in the control group (*p*=0.037) (Table 2). Among the 400 children in clinical remission, there were 153 cases of relapse and 45 cases of nonrelapse in the TCM group and 175 cases of relapse and 27 cases of nonrelapse in the control group. The recurrence rate of children in the TCM group was significantly lower than that of the control group (*p*=0.015) (Table 2).

### 3.3. Relapse-Free Survival

**Figure 1:**
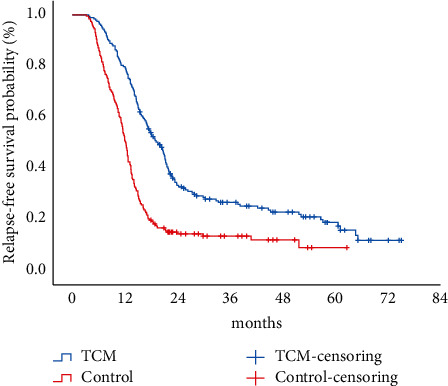
Relapse-free survival between TCM and control groups in children with PNS.

The median relapse-free survival was 19.6 and 12.2 months in the TCM and control groups, respectively. Kaplan–Meier survival curves showed that the median relapse-free survival was significantly shorter in the control group than in the TCM group (log-rank test *p* ¼ 0.000) (Figure 1). In the univariate Cox analysis, TCM was a protective factor for relapse-free survival (*p* ≤ 0.01). Besides, age, clinical classification, and IgM level of the child were also predictors of relapse (*p*=0.038, *p*=0.016, and *p*=0.008). Multifactorial Cox regression showed that TCM was an independent protective factor for relapse-free survival in children with PNS (HR = 0.55, 95% CI 0.49–0.63, *p* ≤ 0.01), while factors such as age, clinical classification, and IgM level were not statistically significant in the multifactorial Cox analysis (*p*=0.572, *p*=0.756, and *p*=0.348) (Table 3). Subgroup analysis of the recurrence risk in children according to the type of Chinese medicine showed that the risk of recurrence was (HR = 0.48, 95% CI 0.35–0.65, *p* ≤ 0.01) for the use of Yuebi Jiazhu Tang, (HR = 0.45, 95% CI 0.33–0.62, *p* ≤ 0.01) for Ganlu Xiaodu Dan, and (HR = 0.60, 95% CI 0.43–0.84, *p*=0.003) for Yupingfeng granules, all of which significantly reduced the risk of recurrence compared with children who did not use Chinese medicine (Table 4).

## 4. Discussion

Some studies have found that adjuvant therapy in Chinese medicine has significant benefits in pediatric primary nephrotic syndrome in recent years. Milkvetch root can increase plasma albumin, reduce urinary protein excretion, decrease serum cholesterol and triglycerides, and improve clinical remission rate in children with PNS with a positive effect on the treatment of adrenal syndrome [11]. In addition, milkvetch root-type formulations of Huaiqihuang, Yupingfeng, and Shenkangling also have good efficacy in improving remission rates and reducing relapse rates [12–14]. However, milkvetch root and milkvetch root-type formulas are mainly applied to children with TCM evidence of Qi deficiency of the lung and spleen, and the effects of other TCM prescriptions in children with PNS have not been reported in the literature. In this retrospective study, the children with PNS in our hospital mainly had three types of syndromes: internal retention of damp-heat, syndrome of fighting of wind with water, and Qi deficiency of the lung and spleen. The symptoms of internal retention of damp-heat were varying degrees of edema, fever and tiredness, stuffiness in the chest and epigastrium, sore limbs and throat, irritable and thirst, or bitter and sticky mouth, or unpleasant stools, red tongue, and yellow and greasy coating, which were treated with Ganlu Xiaodu Dan. The edema of the syndrome of fighting wind with water started from the eyelids, and then, the whole body had different degrees of edema. The onset was rapid, mostly with fever, aching limbs, unfavorable urination, spontaneous sweating, red tongue with white greasy coating, and floating pulse, which were treated with Yuebi Jiazhu Tang. Children with Qi deficiency of the lung and spleen were treated with Yupingfeng granules. The results of the study showed that the formula given according to the TCM evidence could significantly improve the clinical remission rate and reduce the recurrence rate in children with PNS. Meanwhile, this study was the first to investigate the relationship between adjuvant therapy with Chinese medicine and relapse-free survival of PNS, and Chinese medicine could significantly prolong the relapse-free survival of children with PNS and was an independent protective factor for relapse-free children with PNS. The results showed that prescriptions based on TCM syndrome could significantly improve the clinical remission rate and reduce the recurrence rate of PNS children. At the same time, this study for the first time explored the relationship between TCM adjuvant therapy and the recurrence-free survival of PNS children. TCM could significantly prolong the recurrence-free survival of children and was an independent protective factor of PNS children.

Currently, the pathogenesis of PNS is considered to be an inflammatory injury mediated by immune dysfunction. Foxp3+Treg cells were downregulated locally in peripheral blood and the kidney of children with PNS, and IL-17 expression was increased locally in the kidney [15]. IL-17 promotes apoptosis of podocytes and promotes IL-1*β* secretion through activation of the nuclear transcription factor-kB (NF-kB) pathway in human mesangial cells [16]. Immune imbalance of Th17/Treg cells is involved in the development and progression of pediatric PNS, and abnormal activation of the local Th17/IL-17 axis in the kidney is closely related to the sensitivity of PNS to hormone therapy, pathological type, and prognosis [17]. Blackberry lily rhizome and ephedra are the main components in Ganlu Xiaodu Dan and Yuebi Jiazhu Tang, which have been shown to impede Th2/Th17 differentiation, promote CD4+FoxP3+ Treg production, and inhibit NF-kB activity [18]. Baical skullcap root is also a major component in Ganlu Xiaodu Dan. Baical skullcap root can induce Foxp3 expression in T cells, promote Treg cell differentiation and regulatory activity, increase the number of regulatory T cells, and decrease the number of Th1 and Th17 cells [19, 20]. Children with PNS often have a combination of hyperlipidemia, and virgate wormwood herb, as a major component in Ganlu Xiaodu Dan, can reduce blood triglycerides and low-density lipoproteins [21, 22].

## 5. Conclusion

In conclusion, adjuvant treatment of pediatric primary nephrotic syndrome with TCM improved remission rates, reduced relapse rates, and prolonged relapse-free survival and was an independent protective factor against relapse in children with PNS. However, due to the retrospective nature of our study, more prospective randomized controlled studies are needed in the future to further confirm our results.

## Figures and Tables

**Table 1:** Baseline characteristics of the children.

Characteristics	TCM group (*n* = 217)	Control group (*n* = 238)	*P* value
Age	5.05 ± 2.34	4.93 ± 2.25	0.578
Gender (male/female)	164/53	183/55	0.763
Clinical classification (simple type/nephritic type)	178/39	197/41	0.835
24 h urine protein quantification	128.74 ± 52.74	132.65 ± 63.29	0.473
Serum albumin	18.36 ± 4.52	18.74 ± 5.06	0.398
Serum cholesterol	8.57 ± 2.36	8.72 ± 2.58	0.518
Blood creatinine	31.96 ± 11.25	31.37 ± 11.69	0.584
Blood urea nitrogen	4.42 ± 1.87	4.24 ± 1.52	0.263
IgG	3.52 ± 1.44	3.61 ± 1.52	0.517
IgM	1.71 ± 0.78	1.65 ± 0.58	0.356
IgA	1.28 ± 0.61	1.32 ± 0.65	0.499
ESR	55.26 ± 25.43	56.31 ± 26.18	0.665

**Table 2:** Comparison of remission rate and recurrence rate between two groups.

	TCM group	Control group	*P*
Remission	198	202	0.037^*∗*^
Nonremission	19	36	
Relapse	153	175	0.015^*∗*^
Nonrelapse	45	27	

^*∗*^ *P* < 0.05.

**Table 3:** Factors associated with relapse in children with PNS.

Characteristics		Univariate analysis		Multivariate analysis	
		HR (95% CI)	*P* value	HR (95% CI)	*P* value
TCM use		0.47 (0.38–0.59)	≤0.01^*∗*^	0.55 (0.49–0.63)	≤0.01^*∗*^
Age (year)		0.92 (0.85–0.99)	0.038^*∗*^	0.97 (0.93–1.02)	0.572
Gender		1.18 (0.96–1.46)	0.12		
Clinical classification	Simple type	0.71 (0.58–0.94)	0.016^*∗*^	0.85 (0.67–1.03)	0.756
	Nephritic type				
24 h urine protein quantification		1.02 (0.97–1.12)	0.786		
Serum albumin		0.96 (0.82–1.25)	0.931		
Serum cholesterol		1.07 (0.91–1.26)	0.173		
Blood creatinine		1.02 (0.79–1.18)	0.228		
Blood urea nitrogen		1.06 (0.94–1.22)	0.217		
IgG		0.91 (0.74–1.06)	0.577		
IgM		0.83 (0.52–0.94)	0.008^*∗*^	0.95 (0.82–1.09)	0.348
IgA		0.98 (0.78–1.15)	0.870		
ESR		1.01 (0.86–1.31)	0.780		

^*∗*^ *P* < 0.05.

**Table 4:** Risk of relapse according to the use of most common TCM among children with PNS.

TCM		*n*	Recurrence (*n*)	HR (95% CI)	*P* value
Non-TCM group		202	175	1	
TCM group		198	153		
	Yuebi Jiazhu Tang	79	59	0.48 (0.35–0.65)	≤0.01^*∗*^
	Ganlu Xiaodu Dan	67	51	0.45 (0.33–0.62)	≤0.01^*∗*^
	Yupingfeng granules	52	43	0.60 (0.43–0.84)	0.003^*∗*^

^*∗*^ *P* < 0.05.

## Data Availability

The data used to support the findings of this study are available from the corresponding author upon request.

## References

[1] Boyer O., Baudouin V., Bérard E. (2017). Aspects cliniques du syndrome néphrotique idiopathique de l’enfant. *Archives de Pediatrie*.

[2] Lei J., Ma S. (2020). Relationship between TIM-3 gene polymorphisms and steroid-resistant primary nephrotic syndrome in children. *Cellular and Molecular Biology*.

[3] Sandys V., Byrne D. (2016). Acute interstitial nephritis secondary to metamizole; the rise of drug tourism. *Irish Medical Journal*.

[4] Yang F., Jiang X. Y. (2017). Evidence-based guidelines for the diagnosis and treatment of hormone-sensitive, relapsing/dependent nephrotic syndrome in children. *Chin J Pediatr*.

[5] Rajalakshmi S. L., Raman V., Ekambaram S., Subramaniyam G., Gowrishankar N. C., Nammalwar B. R. (2021). Cyclosporine-associated pericardial tamponade in a child with steroid-resistant nephrotic syndrome. *Indian Journal of Pediatrics*.

[6] Couderc A., Bérard E., Guigonis V. (2017). Traitements du syndrome néphrotique cortico-dépendant de l’enfant. *Archives de Pediatrie*.

[7] Wang J., Huang L., Gao P. (2021). Diltiazem on tacrolimus exposure and dose sparing in Chinese pediatric primary nephrotic syndrome: impact of CYP3A4, CYP3A5, ABCB1, and SLCO1B3 polymorphisms. *European Journal of Clinical Pharmacology*.

[8] Toonen E. J. M., Laskewitz A. J., van Dijk T. H. (2014). Glucose kinetics in the collagen-induced arthritis model: an all-in-one model to assess both efficacy and metabolic side effects of glucocorticoids. *PLoS One*.

[9] Tacrolimus B. Y. (2019). 20 years of use in adult kidney transplantation. What we should know about its nephrotoxicity. *Artificial Organs*.

[10] Lin J., Huang L.-M., Wang J.-J., Mao J.-H. (2021). Efficacy and safety of Huaiqihuang granule as adjuvant treatment for primary nephrotic syndrome in children: a meta-analysis and systematic review. *World Journal of Pediatrics*.

[11] Chan E. Y.-h., Webb H., Yu E. (2020). Both the rituximab dose and maintenance immunosuppression in steroid-dependent/frequently-relapsing nephrotic syndrome have important effects on outcomes. *Kidney International*.

[12] Feng M., Yuan W., Zhang R., Fu P., Wu T. (2013). Chinese herbal medicine Huangqi type formulations for nephrotic syndrome. *Cochrane Database of Systematic Reviews*.

[13] Shi X., Zhong X., Ding J. (2018). Adjuvant treatment with Yupingfeng formula for primary nephrotic syndrome in children. *Medicine*.

[14] Zheng J., Ai S., Yang F., Qiu C. X., Lu X. L. (2014). Treatment of senile diseases should prescribe Chinese patent medicine scientifically. *Zhongguo Zhong Xi Yi Jie He Za Zhi*.

[15] Shao X. S., Yang X. Q., Zhao X. D. (2009). The prevalence of Th17 cells and FOXP3 regulate T cells (Treg) in children with primary nephrotic syndrome. *Pediatric Nephrology*.

[16] Wang L., Li Q., Wang L. (2013). The role of Th17/IL-17 in the pathogenesis of primary nephrotic syndrome in children. *Kidney & Blood Pressure Research*.

[17] Free M. E., Falk R. J. (2010). IL-17A in experimental glomerulonephritis: where does it come from?. *Journal of the American Society of Nephrology*.

[18] Lin C.-C., Wang Y.-Y., Chen S.-M. (2020). Shegan-Mahuang Decoction ameliorates asthmatic airway hyperresponsiveness by downregulating Th2/Th17 cells but upregulating CD4+FoxP3+ Tregs. *Journal of Ethnopharmacology*.

[19] Zhu W., Chen X., Yu J. (2018). Baicalin modulates the Treg/Teff balance to alleviate uveitis by activating the aryl hydrocarbon receptor. *Biochemical Pharmacology*.

[20] Bae M.-J., Shin H. S., See H.-J., Jung S. Y., Kwon D.-A., Shon D.-H. (2016). Baicalein induces CD4+Foxp3+ T cells and enhances intestinal barrier function in a mouse model of food allergy. *Scientific Reports*.

[21] Guo Y., Li J. X., Wang Y. L. (2017). Yinchen linggui zhugan decoction ameliorates nonalcoholic fatty liver disease in rats by regulating the Nrf2/ARE signaling pathway. *Evid Based Complement Alternat Med*.

[22] And Alternative Medicine Ec (2019). Retracted: an ErChen and yinchen decoction ameliorates high-fat-induced nonalcoholic steatohepatitis in rats by regulating JNK1 signaling pathway [retraction of: evid based complement alternat med. 2017;2017:4603701]. *Evid Based Complement Alternat Med*.

